# Impacts of testing and immunity acquired through vaccination and infection on covid-19 cases in Massachusetts elementary and secondary students

**DOI:** 10.1038/s43856-024-00619-3

**Published:** 2024-10-16

**Authors:** Westyn Branch-Elliman, Melissa Zeynep Ertem, Richard E. Nelson, Anseh Danesharasteh, David Berlin, Lloyd Fisher, Elissa M. Schechter-Perkins

**Affiliations:** 1 VA Boston Center for Healthcare Optimization and Implementation Research (CHOIR), Boston, MA US; 2 VA Boston Healthcare System, Department of Medicine, Section of Infectious Diseases, Boston, MA US; 3 Harvard Medical School, Department of Medicine, Boston, MA US; 4 School of Systems Science and Industrial Engineering, Binghamton University, State University of New York, New York, NY US; 5 Division of Epidemiology, University of Utah School of Medicine, Salt Lake City, UT US; 6 IDEAS Center, Veterans Affairs Salt Lake City Healthcare System, Salt Lake City, UT US; 7 CIC Health, Cambridge, MA US; 8 Reliant Medical Group, Worchester, MA US; 9 UMass Medical School, Department of Pediatrics, Worchester, MA US; 10 Department of Emergency Medicine, Boston University Chobanian & Avedisian School of Medicine and Boston Medical Center, Boston, MA US

**Keywords:** Epidemiology, Paediatric research

## Abstract

**Background:**

During the 2021–22 academic year, Massachusetts supported several in-school testing programs to facilitate in-person learning. Additionally, COVID-19 vaccines became available to all school-aged children and many were infected with SARS-CoV-2. There are limited studies evaluating the impacts of these testing programs on SARS-CoV-2 cases in elementary and secondary school settings. The aim of this state-wide, retrospective cohort study was to assess the impact of testing programs and immunity on SARS-CoV-2 case rates in elementary and secondary students.

**Methods:**

Community-level vaccination and cumulative incidence rates were combined with data about participation in and results of in-school testing programs (test-to-stay, pooled surveillance testing). School-level impacts of surveillance testing programs on SARS-CoV-2 cases in students were estimated using generalized estimating equations within a target trial emulation approach stratified by school type (elementary/middle/high). Impacts of immunity and vaccination were estimated using random effects linear regression.

**Results:**

Here we show that among *N* = 652,353 students at 2141 schools participating in in-school testing programs, surveillance testing is associated with a small but measurable decrease in in-school positivity rates. During delta, pooled testing positivity rates are higher in communities with higher cumulative incidence of infection. During omicron, when immunity from prior infection became more prevalent, the effect reversed, such that communities with lower burden of infection during the earlier phases of the pandemic had higher infection rates.

**Conclusions:**

Testing programs are an effective strategy for supporting in-person learning. Fluctuating levels of immunity acquired via natural infection or vaccination are a major determinant of SARS-CoV-2 cases in schools.

## Plain language summary

During the height of the Covid-19 pandemic, multiple strategies were used to enable students to participate in in-person elementary and secondary schools. Little is known about the overall impact of prior immunity and in-person testing programs on the ability to maintain protection from Covid-19 in schools. This study, conducted in Massachusetts during the 2021-2022 academic year, found that community immunity gained through prior infection or vaccination, combined with testing strategies including testing programs to monitor infection and test to-stay modified quarantine programs, were safe and effective for allowing in-person learning. These data can be used to shape policy about in-school practices during future respiratory virus pandemics.

## Abstract (web summary)

Branch-Elliman et. al assess the impact of testing programs and immunity on SARS-CoV-2 case rates in elementary and secondary students in Massachusetts. They find that testing strategies are an effective intervention for supporting in-person learning and that immunity acquired from natural infection or vaccination mitigate COVID cases in schools.

## Introduction

During the initial school re-opening in fall 2020, CDC recommendations for mitigating COVID-19 spread in educational settings included indoor masking, testing, and social distancing policies. In March 2021, distancing was de-emphasized, but recommendations about masks and quarantine periods following exposures were maintained^1,2^. Once available, vaccines were promoted as a critical intervention^3^.

Based on these recommendations and national and international evidence^4^, during the 2021–2022 academic year, Massachusetts adopted several COVID-19 mitigation strategies to support in-person learning. These included three types of in-school SARS-CoV-2 testing programs^5–7^: 1) a pooled testing (surveillance) program^8^, 2) a test-to-stay (TTS) modified quarantine program for those exposed to a known positive case during school^9^ and 3) an on-site symptomatic diagnostic testing program. Both the pooled testing program and the TTS program were designed for asymptomatic students. The aim of the pooled testing program was early identification of asymptomatic cases without identified exposures to limit the potential for outbreaks. Recognizing that in-school exposures are lower risk relative to at-home exposures, the aim of the TTS program was to facilitate in-person learning for elementary and secondary school students with identified exposures to SARS-CoV-2 in classroom settings. Given reports from early in the pandemic about risk of spread during early, pre-symptomatic infection when antigen tests might not yet be positive, there were theoretical concerns that TTS programs might lead to increases in-school spread, although there are limited real-world data to estimate additional risk potentially conferred by this strategy.

**Fig. 1:**
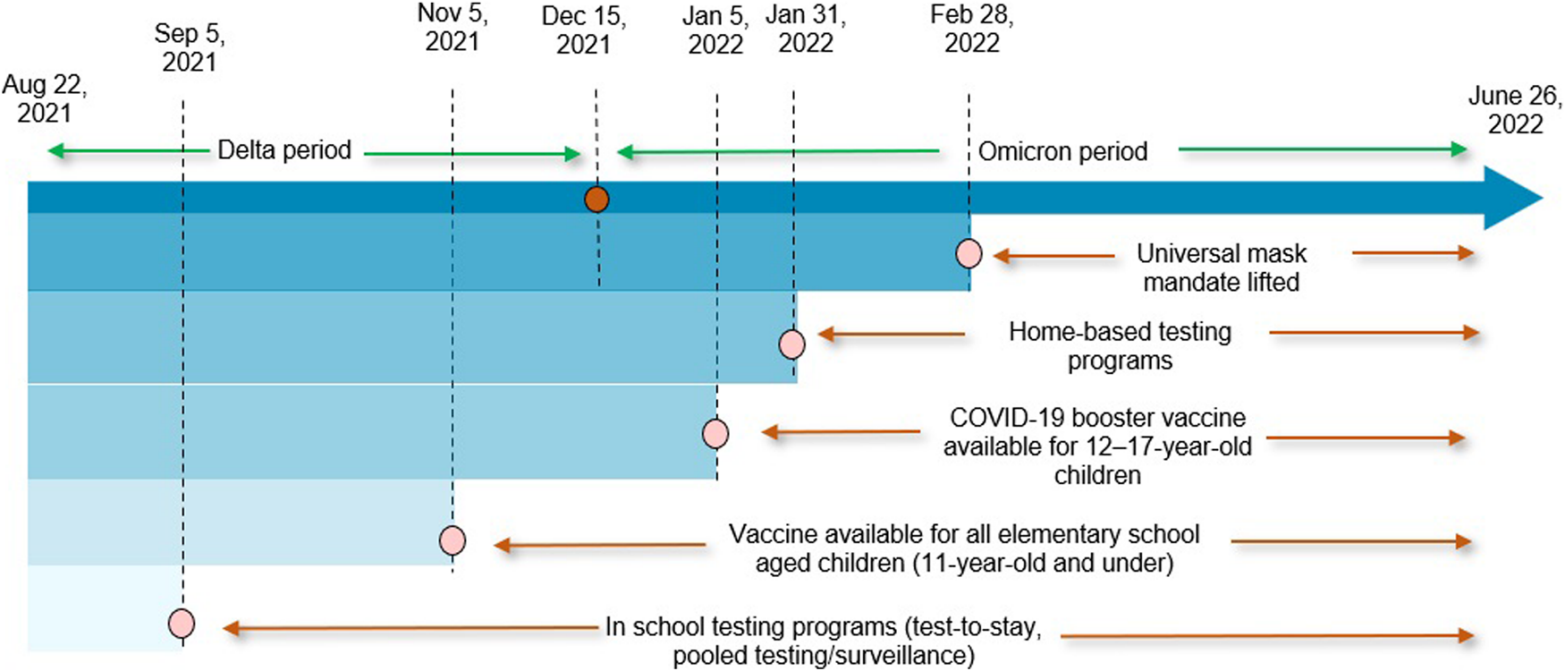
Timeline of Key COVID-19 mitigation policy change milestones in Massachusetts elementary and secondary schools, 2021–2022 academic Year. Key infection mitigation measure policies in Massachusetts elementary and secondary schools throughout the 2021–2022 academic year. Dates and dotted lines indicate date of policy or program implementation or change. Total duration of the policy is indicated with red arrows. Dominant variant is indicated with green arrows.

In addition to the in-school testing programs, a mask mandate was in place at the start of the school year and not lifted until the end of February, 2022 (Fig. 1)^10^. After the state-wide masking mandate was lifted, individual schools and districts made local decisions about masking policy. COVID-19 vaccinations were not mandated but encouraged. Effective November 2021, schools with greater than 80% vaccination rates were eligible to apply for a waiver to lift the mask mandate; during the fall semester, this waiver only applied to high schools as the vaccine was not yet approved for individuals under 12 years of age^11^.

Longitudinal, state-wide data about in-school mitigation measures, test results, and program participation were combined with state-wide data about community case rates and vaccination rates at the town-level to evaluate the central objective of this state-wide retrospective study: to estimate the impact of mitigation measures (e.g., vaccination rate, immunity from prior infection, testing programs) on student COVID-19 case rates.

Pooled testing programs are associated with a trend toward a small but measurable decrease in SARS-CoV-2 cases in schools. Immunity, either from vaccination or natural infection, is an important mitigator of cases with waxing and waning impacts.

## Methods

### Overview

A state-wide longitudinal cohort at the biweekly level during the period from 9/5/2021-6/26/2022 was created by combining: testing data and data about school populations collected by the Massachusetts Department of Elementary and Secondary Education (DESE), vaccination and town-level community COVID-19 case rates from the Massachusetts Department of Public Health^12^, and data from the National Center for Education Statistics (NCES)^13^ about school type and demographics. Consent rates for the testing programs were obtained from CIC Health^6^, the private public health company that partnered with the state to facilitate in-school testing during the 2021–22 academic year.

Key policy milestones that occurred throughout the academic year included: testing program availability at the start of the academic school year, vaccine availability to 5-11-year-olds on 11/2/2021, switch to decreased state support for TTS (with a pivot to distribution of weekly at-home antigen tests) on 1/31/2022 and lifting of the state-wide in-school mask mandate on 2/28/2022 (Fig. 1).

### Testing programs & consent process

During the 2021-2022 academic year, three different optional testing programs were available to students and staff in all Massachusetts schools (Table 1)^5,14^. First, a weekly pooled testing (surveillance) program^8^ in which samples from 2–10 individuals were pooled and tested using PCR to identify positive cases. If a positive pool was identified, additional testing was undertaken to identify the positive case among the pool; thus, individuals included in the pool were tested twice, often resulting in multiple positive tests for each positive individual. The pooled testing program was designed for longitudinal repeated testing. Most commonly, individuals participating in this program underwent pre-planned weekly testing. Because testing was pre-planned, repeated, and independent of exposures or other policies, pooled testing positivity results provide information about case rates over time that is not directly impacted by other policies or testing based on contact tracing. Vaccinated students were not excluded from participation.

**Table 1:** Details of In-school testing programs

Testing Program	Eligible Individuals	Testing Frequency	Duration
Pooled testing (surveillance)	All asymptomatic individuals (students and staff)	Weekly planned repeated testing*	Academic year
Test-to-stay	All asymptomatic individuals (students and staff)	As-needed based on identified in-school exposures	Start of academic year; program optional effective 1/31/2022; program adapted to move to distribution of testing kids in school for voluntary at-home testing
Symptomatic testing	Any individual on a school campus with potential SARS-CoV-2 symptoms	As-needed based on symptoms	Academic year

The second testing program was a TTS modified quarantine program for asymptomatic students meeting criteria for in-school COVID-19 exposure^9^. Rather than automatically being excluded from school, as they might otherwise have been given national guidelines concerning exposures at that time, these students took daily antigen tests on site at the beginning of the school day, and, if negative, continued to participate in in-person learning. At the start of the program, per contemporaneous CDC guidance, in-school exposure was defined as: within 3 feet of distance for >15 min if both masked, or 3–6 feet of distance if 1 or both unmasked. Also per contemporaneous CDC guidance, vaccinated students were excluded from the close contact definition. If students or staff declined to participate in the TTS program, or if TTS was not available in their district, then a 10-day at home quarantine period was recommended. Individual districts and schools made determinations about referrals to and participation in testing programs based on local contact tracing efforts and determination of in-school exposures and thus decisions about testing vaccinated contacts were made at the school level. During the active phase of the program, only in-school exposures were eligible for participation in TTS; exposures that occurred outside of classroom settings were recommended to undergo a 10-day quarantine period. On-site antigen symptomatic testing was a third type of testing program, available for individuals who became sick during the school day.

Effective 1/31/2022, due to ongoing frequent exposures in and out of the classroom, straining contact tracing resources, there was a state-wide shift away from the TTS program to an at-home weekly self-testing program with distribution of antigen tests at school^5^. The pooled testing program was available for the duration of the school year.

School districts and individual schools selected which testing program or combinations of three programs were locally offered and available; within a school, anyone interested in participating in the program had the option to opt-in. Individual participants were *only* offered the option of enrolling in all the testing programs that were offered by the district; there was not the option to selectively enroll in testing programs (e.g., one could not consent to test-to-stay but not surveillance testing). Parental or legal guardian consent for participation was required for minor participants and was obtained via both paper and electronically. Participants were able to opt-in and opt-out of the programs throughout the school year.

### Cohort creation

A longitudinal dataset at the bi-weekly level for schools nested within towns was created (Supplementary Table 1, Data Sources). School-level COVID-19 testing and consent data were obtained from CIC Health^6^. Key variables included: proportion of students in the school consented to participate, total number of tests performed, type of test performed, total number of tests positive, total number of surveillance pools performed, total number of pools positive, and information about reflex testing results. For antigen testing, the age of the individual tested was available. Reflex test results, which represent repeated testing, were excluded to avoid double-counting of cases. For pooled testing results, because the pools contained multiple individuals, age was not calculable. Percentage of consented students was calculated by dividing the number of individuals participating in the program by the total school population, which, along with information about school type and demographics, was obtained from DESE and NCES^15^. Limited individual demographic data were available, so individuals ≤19 years of age were classified as students and individuals >19 years of age were classified as staff members. Schools were excluded if they were missing consent data, typically due to paper consenting processes or if they could not be matched to a CIC Health-test results file, which occurred in approximately 2% of all schools state-wide, primarily due to utilization of a separate tracking system. Data linked to the district only and not a specific school were also excluded.

Cumulative town-level SARS-CoV-2 case rates per 100,000 residents and vaccination rates stratified by age group on a biweekly basis were collected from the Massachusetts Department of Public Health^16,17^. Town-level cumulative prior infection and vaccination rates were both included as measures of immunity.

### Outcome

The school-level testing program initiation date was defined as the date of the first test result documented in the system.

#### Impact of pooled testing program

To assess the impact of pooled testing, the primary outcome was school-level biweekly test-to-stay positivity rate, defined as the number of positive test-to-stay tests divided by the consented population. The secondary outcome of the first analysis was school-level total test positivity rate, defined as the total number of positive tests (pooled tests, test-to-stay tests, symptomatic tests) divided by the consented school population.

#### Impacts of vaccination and immunity

To assess the impact of vaccination and immunity, the primary outcome was school-level biweekly surveillance testing positivity rate, defined as the number of positive surveillance pools divided by the consented population. All test positivity rate was evaluated secondarily.

### Exposures

The exposure variables evaluated were different in-school mitigation factors. The impact of the pooled testing program was evaluated as a dichotomous variable, with the primary outcome assessed the TTS positivity rate. TTS positivity was selected as the primary outcome because, in theory, early identification of asymptomatic cases through the pooled testing program would reduce onward transmission to exposed classmates, therefore reducing the positivity rate among identified in-school exposures. Impact on all-test positivity rate, which included pooled testing results, was assessed secondarily. Due to near universal participation, the TTS program is presented descriptively.

Assessments of the impact of vaccination rates were performed in two ways. First, school-level vaccination rate was modeled as a continuous variable and assessed in multivariate models with pooled testing positivity rate as the primary outcome. Pooled testing positivity rate was selected as the primary outcome for the impact of vaccination and prior immunity analysis because these tests were collected independent of exposure, unlike TTS. Exposures at the time that determined referral to the TTS program were defined in part by vaccination status. Second, schools were classified into high (> 80%) vaccination or low (< 50%) vaccination uptake, and pooled testing positivity rate was estimated as the primary outcome. For high schools, high vaccination uptake was defined based upon receipt of at least two doses of vaccine on or before 1/1/2022. For elementary schools, where vaccines were not available until 11/2021, high vaccination uptake was defined based upon receipt of at least one dose of vaccine on or before 1/1/2022. Given vaccine availability, impact of vaccination was evaluated in elementary and high schools during the omicron period before and after lifting of the mask mandate (e.g., before and after 2/28/2022). To evaluate the impact of immunity acquired from prior infection, the association between existing cumulative town-level (e.g., community) incidence rate and in-school positivity rate was assessed and stratified by circulating variant (delta, omicron).

### Covariates

Additional independent variables included in regression models were town-level vaccination rates. School-level variables included: level of school (elementary, middle, high, mixed age), school type (public, private, charter), school size, and school demographic data (student race and ethnicity, Title I status, rurality), obtained from NCES.

### Statistics and reproducibility

#### Surveillance testing program impact

The impact of surveillance testing on TTS positivity rate was estimated using a matched retrospective observational cohort with a target trial emulation approach, a causal inference method in which observational data are used to “mimic” a randomized controlled trial. Details of the emulation approach are presented in Table 2. Massachusetts schools with a testing program implemented during the 2021-22 academic year were potentially included. Schools were excluded if they initiated surveillance testing prior to 9/19/2021.

**Table 2:** Matched observational retrospective cohort with a target trial emulation design

Protocol Component	Description
Eligibility criteria	• Massachusetts schools with testing program implemented during the 2021-22 school year • No surveillance testing program before 9/19/2021
Policy interventions	• Intervention: Implementation of in-school surveillance testing program on 9/19/2021 • Control: No surveillance testing program
Follow-up period	• 12 weeks after 9/19/2021 (study end: 12/12/2021)
Matching criteria	• Massachusetts schools that did not have a surveillance testing program on the study start date were propensity-matched 1:1 based on average vaccination at the level of the school-aged population, consent rate, race, urban/rural, title1 status, charter, masking policy, public/private
Outcome(s)	• Primary: In-school test-to-stay positivity rate • Sensitivity analysis: In-school all test positivity rate
Critical co-variates	• Community cases per 100,000 at the town-level, Treatment/control, weeks from program start
Causal contrasts of interest	• Intention-to-treat*:* Once included in the study, all schools were analyzed according to their initial assignment (intervention or control).
Statistical analysis	• Primary: cluster GEE model adjusted for critical co-variates • Secondary: Fixed effects model • Sensitivity analysis: Counties that implemented surveillance testing after the study start date were excluded

Schools that initiated a surveillance testing program on 9/19/2021 were then propensity-matched 1:1 to any school meeting eligibility criteria that did not have a surveillance testing program implemented by 9/19/2021. Propensity matching was based on average vaccination rate in the school population at the town-level, in-school consent rate, race, urban/rural status, title I status, school type (e.g., charter, public, private). The biweekly difference in TTS positivity rate comparing schools with and without pooled testing programs was then assessed using the following multivariable regression specification estimated with generalized estimating equations (GEE) with a gaussian family and an identity link function:

$${{{\rm{E}}}}\left({{{{\rm{y}}}}}_{{{{\rm{it}}}}}\right) = \,{{{{\rm{\beta }}}}}_{1}({{{\rm{Treatment}}}})_{{{{\rm{it}}}}}+{{{{\rm{\beta }}}}}_{2}\left({{{\rm{Weeks}}}}\; {{{\rm{from}}}}\; {{{\rm{program}}}}\; {{{\rm{initiation}}}}\right)_{{{{\rm{it}}}}} \\ + \,{{{{\rm{\beta }}}}}_{3}\left({{{\rm{Proportion}}}}\; {{{\rm{vaccinated}}}}\right)_{{{{\rm{it}}}}}+\,{{{{\rm{\beta }}}}}_{4}({{{{\rm{Community}}}}\; {{{\rm{cases}}}}})_{{{{\rm{it}}}}}$$

where the dependent variable, *y*_*it*_, is the in-school TTS positivity rate (positive tests/school-level consented population/biweekly period) in school *i* at time *t*. For the secondary analysis, the dependent variable was the all-test positivity rate in school *i* at time *t*. All test positivity rate includes results from all TTS and pooled tests performed in school *i*. The independent variable of interest, *Treatment*_*it*_, is an indicator for the presence of a pooled testing program. We also included covariates measuring the number of weeks since 9/19/2021 (*Weeks from program initiation*), the school vaccination rates (*Proportion vaccinated*), and the SARS-CoV-2 case rate in the surrounding town (*Community cases*). The analysis for estimated Eq. (1) was completed using an intention-to-treat approach.

### Assessments of the impact of vaccine and natural immunity

The impacts of immunity, vaccination, and testing program availability on pooled testing positivity rates at the school-level were estimated using random effects linear regression adjusted for district demographic variables and stratified by school type (elementary/middle/high):

1 $${y}_{i,t}=\,{\beta }_{0}+\,{\beta }_{1}{X}_{i,t}+\,{\mu }_{i}+\,{v}_{i,t}+\,{\epsilon }_{t}\,$$

Where the dependent variable, y_i,t_, represents the in-school pooled testing positivity rate or all test positivity rate (positive tests/school-level consented population/biweekly period) in school i at time t in as the primary and secondary outcome, respectively. All test positivity rate includes results from all TTS and pooled tests performed in school *i*.

Due to vaccines being available at different times for different age groups of children (5/18/2021 for ages 12–17 and 11/5/2021 for ages 5–11) and inherent differences in in-school contact patterns, the linear regression models were stratified by school level (elementary, middle, high, mixed grade levels). Additionally, given the scale-up of the program during the early weeks of the school year, we conducted an additional sensitivity analysis in which the first 6 weeks of the school year were excluded to test the robustness of study findings.

All data were analyzed using STATA v.17.

### Ethics approval and consent to participate

This study was approved by the VA Boston Research and Development Committee as non-human subjects research prior to data collection and analysis.

## Results

### Cohort creation

Among *N* = 2304 Massachusetts schools, *N* = 163 schools were excluded due to difficulty retrieving consent data, typically due to paper consenting processes. Of the remaining 2141 schools (1523 public, 618 private), an estimated 1,100,199 students were included. There were 987 elementary schools, 303 middle schools, 356 high schools, and 495 mixed schools (Table 3). Approximately 59% Kindergarten to 12th grade (K to 12) students consented to participate in testing programs (652,353/1,100,199). School-level consent rates among elementary and mixed schools (for which vaccination was not available for the majority of students until 11/2021) were positively correlated with vaccine uptake; in middle and high schools (for which vaccines were available for most students prior to the start of the academic year), school-level consent rates were negatively correlated with vaccine uptake (Supplementary Fig. 1).

**Table 3:** Descriptive statistics of the participants in the state-wide in-school testing programs

	All participating schools	Surveillance testing available	Test-to-stay testing available
Total number of schools	2141	1573	2006
Total school population	1,100,199	814,876	1,059,212
Number of students consented (%)	652,353 (59%)	496,144 (61%)	614,872 (58%)
Elementary* (age <12)	N/A	N/A	333,063
Middle* (12 <= age <15)	N/A	N/A	177,781
High* (15 <= age <19)	N/A	N/A	90,802
School Type			
Public	1521	1108	1458
Private	620	465	548
Demographics of enrolled students			
White	58.36	56.05	57.43
Black	8.6	9.68	8.83
Other	13.04	12.9	13.74
Hispanic	20	21.37	20
Demographics of schools (%)			
Title 1 status	20.9 (449)	22 (346)	21.2% (425)
Rural status	8.3 (177)	8.84 (139)	8.52% (171)
Charter	2.4 (51)	2.73 (43)	2.19% (44)
Staff participation in testing programs	32,648	12,134	6933

Data collected from the Massachusetts Department of Elementary and Secondary Education and from the National Center for Education Statistics. Table includes data on the number of schools participating in teach type of testing program, the total number of consented students stratified by their school type, and demographic data about enrolled students, including data about race and ethnicity (% White, % Black, % Hispanic, % Other). Title 1 status was obtained from national and state databases and is defined based on the percentage of children from low-income families within a school setting.

*Given the nature of the available data, only age ranges are reported. Elementary school-age was defined as <12 years old; Middle school-age was defined as ≥12 years old to <15 years old; High school-age was defined as ≥15–19 years old.

### Testing programs: participation and program scope

**Fig. 2:**
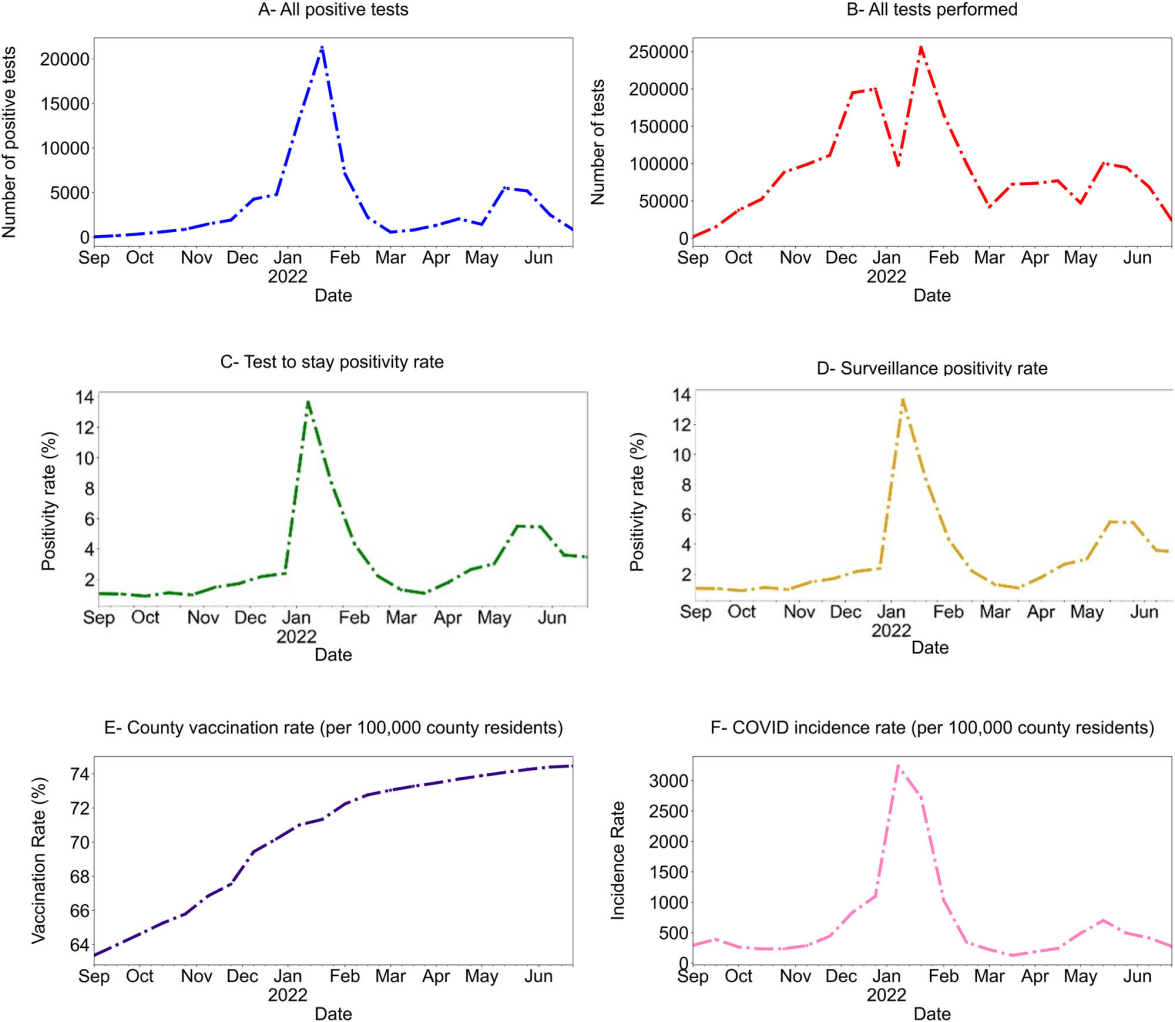
Unadjusted mean in-school test positivity rates and testing density, county-level vaccination and incidence rates: Massachusetts, September 2021– June 2022. Panel **A**: unadjusted means for total number of COVID-19 positive tests identified as part of the state-wide testing program (includes all test types). Panel **B**: Total number of COVID-19 tests performed over the course of the 2021-2022 academic year (includes all test types). Panel **C**: Test-to-stay positivity rates. Panel **D**: Pooled testing (surveillance) positivity rate. Panel **E**: County-level vaccination rate, presented as per 100,000 county residents. Panel **F**: County-level SARS-CoV-2 incidence rate per 100,000 county residents.

A total of 2141 schools participated in testing programs (1573 surveillance, 2006 TTS); approximately 764,484 students participated in surveillance testing and 1,053,236 in TTS (non-unique consents). *N* = 1,977,748 tests were completed during the 23 bi-weekly study periods (1,069,232 pooled samples, 690,239 TTS tests, and 218,277 other tests, Supplementary Table 2). Each pool contained 2–10 individual’s samples, with a mean of 5.03 samples. Thus, the pooled tests represented approximately 5,378,237 tests performed on participants during the year-long program. Vaccination rates and case rates are presented in Supplementary Fig. 2 and Supplementary Table 3. Trajectory of case rate changes tracked closely with changes in community case rates (Fig. 2, Supplementary Fig. 3).

The overall positivity rate for the TTS program during was 2% (2700/154,333), with a range of 0.61% during the fall of 2021 to a maximum of 5.63% during the first omicron peak (Supplementary Table 3); trends in TTS positivity closely tracked with community incidence rates (Supplementary Fig. 4). The maximum number of TTS tests performed during the active phase of the program was 139,400 and occurred during the biweekly period starting on 12/26/2021 and including the return from winter vacation. Overall, the program is estimated to have saved at more than 750,000 in-person school days prior to the policy change toward distribution of tests for at home use.

### Testing program impacts

451 schools that initiated a pooled testing program on 9/19/2021 were propensity-matched to 451 schools that did not have a pooled testing program (Table 4). Intervention and control schools were generally similar but intervention schools had slightly higher participation rates, slightly lower proportions of white students, were more likely to be urban than rural, and had higher participation from Title I schools.

**Table 4:** Characteristics of propensity-matched schools with and without pooled testing programs

	Schools with pooled testing	Control schools
Total number of schools	451	451
Total school population	239,161	229,327
Number of students consented (%)	142,409 (59%)	132,111 (57%)
School type		
Public	332	336
Private	119	115
Demographics of enrolled students		
White	53.44%	61.74%
Black	8.09%	7.08%
Other	11.02%	13.46%
Hispanic	27.45%	17.72%
Demographics of schools (%)		
Title 1 status	27.49% (124)	20.18% (91)
Rural status	5.54% (25)	8.87% (40)
Charter	1.77% (8)	1.77% (8)

Over the 12-week period evaluated, elementary, middle, and combined schools that initiated pooled testing had lower TTS positivity rates compared to schools that did not have these programs (Table 5). Effect estimates for TTS positivity ranged from no effect in high schools to a 3% reduction in mixed- school settings. Trends for all-test positivity rates were similar, but this was statistically significant only for mixed school settings (Supplementary Table 4). Results were similar when the first six weeks of the school year were excluded.

**Table 5:** Impact of pooled testing program on test-to-stay positivity rates among propensity-matched schools with and without pooled testing programs

	Elementary	*P*-value	Middle	*P*-value	High	*P*-value	Combined Program	*P*-value
Pooled testing program (Yes/no)	−0.016 [−0.03, −0.005]	0.004	−0.01 [−0.01, −0.003]	<0.001	−0.03 [−0.07, 0.012]	0.179	−0.03 [−0.06, −0.002]	0.036
Town-level case rates (per 100,000 county residents)	−0.0000002 [−0.0000004, 0.00000002]	0.081	−0.0000001 [−0.0000003, 0.0000001]	0.210	−0.000001 [−0.000003, 0.000001]	0.322	−0.0000003 [−0.000001, 0.000001]	0.648
In-school vaccination rate	−0.008 [−0.02, 0.002]	0.102	−0.003 [−0.01, −0.001]	0.007	−0.01 [−0.03, 0.01]	0.317	0.001 [−0.03, 0.03]	0.944

Effects estimated using general estimating equations. Pooled testing program start date: 9/19/2024. Variables used for propensity matching (average vaccination, consent rate, race, urban/rural, Title 1 status, charter, and public/private status) not included in the regression models. For the propensity adjustment, average vaccination rate for the whole population was used. Models are adjusted for age-stratified vaccination rates and program week. Variables in bold are statistically significant to *p* < 0.05. Wald test was used to evaluate statistical significance.

Elementary schools: *n* = 235,546 independent COVID-19 tests were performed, estimated effect size: 187.81. Middle schools: *n* = 64,165 independent COVID-19 tests were performed, estimated effect size: 167.18. High schools: *n* = 48,404 independent COVID-19 tests were performed, estimated effect size: 144.69. Combined schools: *n* = 136,626 independent COVID-19 tests were performed, estimated effect size: 256.84.

### Impacts of immunity from prior infection and vaccine uptake

In the random effects linear regression model, cumulative town prior infection rate, a measure of prior disease burden and population natural immunity, was significantly associated with in-school testing positivity rates (both pooled testing positivity rates and all-test positivity rates), with variable effects depending on predominant variant and acquired immunity. During the Delta period, when immunity from prior infection was low, lower historical cumulative community (town-level) burden of infection was associated with lower SARS-CoV-2 cases in schools (Supplementary Tables 5 & 6). During the Omicron period, lower historical cumulative community burden of prior infection was associated with higher SARS-CoV-2 cases in schools.

Unadjusted mean SARS-CoV-2 test positivity rates in high versus low vaccine uptake elementary and high schools are presented in Supplementary Figs. 6 & 7. After multivariable adjustment, during the period from 1/1/22-2/28/22, elementary schools with high vaccination rates had a 5.1% lower pooled testing positivity rate than schools with low vaccination rates (Table 6). After March 1st, the impact of high vaccination dissipated, and schools with lower vaccination rates had 0.5% lower pooled testing positivity rates than high-vaccination schools. In high schools, high vaccination uptake was associated with a 3.9% reduction in pooled testing positivity rate versus schools with <50% vaccination rates, and findings were similar throughout the year and in sensitivity analyses (Supplementary Tables 7 & 8).

**Table 6:** Impact of high versus low vaccination uptake and cumulative community incidence on pooled testing positivity rates in elementary and high schools during the omicron period

		Elementary schools				High schools			
		β-coefficient	p-value	Confidence interval		β-coefficient	*p*-value	Confidence interval	
Jan 1st – Feb 28th	Cumulative community incidence rate (per 100,000 town residents)	−2.085	<0.001	−2.160	−2.009	−2.530	<0.001	−2.635	−2.424
	Consent rate	−0.069	<0.001	-0.085	−0.054	−0.088	<0.001	−0.110	−0.067
	*Student Demographics*								
	African American	−0.0002	0.503	−0.001	0.0001	0.001	0.005	0.000	0.002
	Hispanic	0.001	<0.001	0.001	0.001	0.002	<0.001	0.001	0.003
	White	−0.0002	0.363	−0.001	0.000	0.001	0.033	0.000	0.001
	Other	0.0001	0.584	0.000	0.001	-0.0001	0.977	-0.001	0.001
	*School Demographics*								
	Rural status	−0.039	<0.001	−0.050	−0.027	−0.040	0.002	−0.066	−0.015
	Title 1 status	0.009	0.028	0.001	0.018	0.031	0.001	0.012	0.050
	Charter school	−0.001	0.968	−0.050	0.048	0.019	0.220	−0.011	0.048
	Public school	0.003	0.612	−0.008	0.014	-0.053	<0.001	−0.070	−0.036
	Low vaccination ( < 50%)*	0.051	<0.001	0.041	0.060	0.039	<0.001	0.021	0.056
Mar1st– June 26	Cumulative community incidence rate (per 100,000 town residents)	0.217	<0.001	0.200	0.234	0.311	<0.001	0.279	0.342
	Consent rate	0.005	0.001	0.002	0.007	0.009	<0.001	0.004	0.014
	*Student Demographics*								
	African American	0.0001	0.111	0.000	0.000	−0.0001	0.816	0.000	0.000
	Hispanic	−0.0001	0.006	0.000	0.000	−0.0001	0.010	0.000	0.000
	White	0.00004	0.191	0.000	0.000	0.0004	0.333	0.000	0.000
	Other	−0.00004	0.298	0.000	0.000	0.0004	0.480	0.000	0.000
	*School Demographics*								
	Rural	0.004	<0.001	0.002	0.006	0.001	0.761	−0.005	0.006
	Title 1 status	−0.001	0.122	−0.003	0.000	−0.004	0.020	−0.008	−0.001
	Charter school	0.001	0.874	−0.007	0.008	−0.002	0.508	−0.008	0.004
	Public school	0.000	0.617	−0.002	0.001	0.007	<0.001	0.004	0.011
	Low vaccination ( < 50%)*	−0.005	<0.001	−0.006	-0.003	-0.008	<0.001	-0.012	−0.005

Effect estimates generated using random effect linear regression. Pooled testing positivity rate estimated outcome. Wald test was used to evaluate statistical significance. Variables in bold are significant to *p* < 0.05.

*Estimated based upon the proportion of individuals in different age brackets vaccinated within a town.

School-specific data were not available for analysis. For high schools, vaccination level was defined as receipt of two-doses by 1/1/2022. *n* = 232,896 COVID-19 tests were performed, estimated effect size: 1392.06. For elementary schools, vaccination level was defined as receipt of one dose by 1/1/2022. *n* = 719,167 COVID-19 tests were performed, estimated effect size: 1863.23.

On 2/28/2022, lifting of the state-wide masking mandate was announced. Prior to 2/28/2022, *N* = 73 schools included in this analysis applied for a waiver of the mandatory masking policy and *N* = 68 were approved. Date about mask lifting was available for *N* = 64 schools. Of these, *N* = 6 lifted their masking mandate prior to 1/1/2022 and maintained the lifting; *N* = 18 shifted to masking optional after 1/15/2022, *N* = 5 switched between masking optional and masking required, and *N* = 35 applied for the waiver but did not lift masking requirements in schools until after the state-wide mandate was lifted.

## Discussion

Our analysis using a year-long, state-wide cohort with granular data about in-school testing programs, participation, mitigation policies, and community vaccination and cumulative case rates advances understanding about the effectiveness of different COVID-19 mitigation strategies in elementary and secondary school settings. To our knowledge, ours is the first to assess the impact of the scope of in-school testing programs and immunity acquired via vaccination and through natural infection on COVID-19 case rates in school settings. Using data from nearly 2 million pooled testing and antigen tests performed, we found in-school pooled testing (surveillance) programs are associated with a trend toward a small but measurable decrease in school-wide infection rates, and that immunity, whether acquired via vaccination or prior infection, was an important mitigating factor with waxing and waning impacts.

The TTS program saved an estimated 750,000 school days during the 5-month period prior to the transition to home-based testing. When TTS programs were implemented, there was concern that allowing exposed students to continue to attend in-person school would lead to increases in in-school transmission due to pre-symptomatic spread. Reassuringly, in this state-wide analysis, we found that the positivity rate in TTS programs was low and tracked closely with community case rates; the peak TTS positivity rate coincided with the peak of the first omicron wave in the community. The low TTS rate is reassuring that the program did not cause substantial forward transmission in school settings. However, although the TTS program was safe and effective for supporting in-person learning, implementing the program was challenging, particularly during periods of high community incidence. The TTS strategy was functionally limited by the amount of contact tracing required to support the program. During the first Omicron wave, ongoing, frequent exposures inside and outside of the classroom created feasibility challenges due to limited resources to measure and track exposures. At times, greater than 50,000 tests were performed per week, a major challenge for school settings, which are not generally equipped to provide medical services. Thus, policy adaptations (specifically, a transition to distribution of tests in school with administration at home) were necessary as the program was no longer sustainable in its original form.

Surveillance testing programs were associated with trends toward small decreases in both all test and TTS positivity rates, however, this came at the expense of a high volume of negative tests number needed to prevent one case. These findings suggest testing programs that limit at-home quarantine and support in-person education should be prioritized when other more sustainable interventions are not yet available.

Findings about the safety of the testing programs are well aligned with other recent work evaluating transmission risk. Tayyar et al. found that most asymptomatic individuals, even with a positive PCR test, have minimal potential to spread to others^18^. Because most asymptomatic people, even with a positive PCR test, do not have significant transmission potential, the overall impact of the surveillance testing program was limited. Additionally, data suggest that the majority of transmissions occur after greater than one hour, and that household contacts account for 40% of all infections^19^. In school, most classes last less than an hour and thus most exposures of short enough duration so as to confer minimal risk of transmission.

High vaccination uptake was protective in elementary schools during the Omicron wave, but impacts dissipated after approximately 8–10 weeks. Waning of protection against infection was not identified in high schools, however, this finding is likely impacted by the approval and administration of booster doses in this population in early 2022, which likely caused a transient protection against infection^20^. Chemaitelly et al. found that pediatric and adolescent vaccination series were associated with a short-term decrease in susceptibility to any infection, but that protection wanes quickly, which aligns with our findings^21^. Other studies in other populations have suggested that immunity obtained via vaccination, prior infection, or both can reduce the risk of subsequent infection by 20–40%, also consistent with our study^22^.

Before the Omicron period, most of the population had not yet acquired immunity, therefore higher cumulative community case rates were associated with substantially higher case rates in schools. During the second half of the school year, higher prior cumulative community case rates were associated with substantially *lower*-case rates, suggesting a role of natural and hybrid immunity in mitigating in-school positivity. We are not able to attribute causality of these findings, which may be due in part to other factors that occurred concurrently, such as lifting of the state-wide masking mandate and different dominant variants. However, there appears to be no similar change in high schools at the time when mask mandates were lifted, suggesting that the finding is likely to be driven at least in part by population immunity acquired through vaccination, natural infection, or both.

Use of pooled testing positivity among the consented population as the primary outcome of interest for evaluating vaccination and immunity impacts is a major strength of the study, as pooled (surveillance) testing positivity is not impacted by definitions of in-school exposure, nor biases in testing rates that may be present in other studies. To ensure robustness of study findings, we also evaluated the impact of different mitigation measures on all positive test results and findings were similar. Another strength of our large and comprehensive state-wide study is the scope and variety of data included in our analysis; we considered in-school mitigation measures and school-level co-variates, community variables, and included granular data about participation rates and consent rates.

This study has several limitations. This was a retrospective observational cohort, not a controlled trial and thus residual confounding is possible, even with a target trial emulation approach. We were only able to access aggregate, de-identified data. This has several implications. Duplicate consents could not be entirely removed, thus actual consent rate may be lower than is estimated. Due to students opting in and out, we were not able to ascertain how participation varied throughout the school year. Additionally, we did not have access to all paper-signed consents. Paper consenting was more common during program scale-up; to assess potential impact, sensitivity analyses were completed with the first six weeks of the school year excluded and estimates did not change. We had access to county-level case rate and town-level vaccination rates, but not district or school level rates, which may have caused some misclassification, particularly for private schools where enrolled students may attend school and live in different counties. Although it is possible that masking policy differences in different districts impacted findings about vaccination and immunity, it is unlikely that these policy differences substantially impacted results prior to 2/28/2022 because of the limited number of schools that relaxed masking policies during this period (33 schools/ 2141, 1.54% of the study cohort). Finally, we only had access to test results entered into the primary tracking system; approximately 2% of schools used a different system and results were not available for analysis. However, it is unlikely that inclusion of this small number of schools would substantially change findings.

## Conclusions

Over the course of a year-long, state-wide testing program, almost 600,000 unique students participated, and nearly 2 million tests were performed. Surveillance testing programs were associated with a small but measurable decrease in school case rates. Waxing and waning population immunity appeared to be an important mitigating factor. Findings can be used to inform future policy about in-school mitigation of respiratory viruses, particularly about implementation of testing programs and timing of vaccination campaigns.

## Supplementary information

Supplementary Materials

## Data Availability

Information about source datasets is included in the Supplementary Materials (Supplementary Table 1). A file with the linked publicly available source data are available for download in the Harvard Dataverse: (https://dataverse.harvard.edu/dataset.xhtml?persistentId=doi:10.7910/DVN/HBK8Q8)^23^. Data from the Department of Elementary and Secondary Education (DESE)/CIC Health is not publicly available but can be obtained following written requests for access to DESE and confirmation of approval, followed by a written request to the corresponding author.

## Code availability

Code underlying analyses are available at the Harvard Dataverse (https://dataverse.harvard.edu/dataset.xhtml?persistentId=doi:10.7910/DVN/HBK8Q8)^23^.

## References

[1] van den Berg, P. et al. Corrigendum to: Effectiveness of 3 Versus 6 ft of Physical Distancing for Controlling Spread of Coronavirus Disease 2019 Among Primary and Secondary Students and Staff: A Retrospective, Statewide Cohort Study. *Clin. Infect. Dis.*10.1093/cid/ciab1049 (2022). 35170734 10.1093/cid/ciab1049 PMC9125402

[2] van den Berg, P. et al. Effectiveness of 3 Versus 6 ft of Physical Distancing for Controlling Spread of Coronavirus Disease 2019 Among Primary and Secondary Students and Staff: A Retrospective, Statewide Cohort Study. *Clin. Infect. Dis.* **73**, 1871–1878 (2021). 33704422 10.1093/cid/ciab230 PMC7989511

[3] US Centers for Disease Control and Prevention Control. *Operational Guidance for K-12 Schools and Early Care and Education Programs to Support Safe In-Person Learning*, https://www.cdc.gov/coronavirus/2019-ncov/community/schools-childcare/k-12-childcare-guidance.html (2022).

[4] Schechter-Perkins, E. M., van den Berg, P. & Branch-Elliman, W. The Science Behind Safe School Re-opening: Leveraging the Pillars of Infection Control to Support Safe Elementary and Secondary Education During the COVID-19 Pandemic. *Open Forum Infectious Diseases* **9**, 10.1093/ofid/ofab134 (2021). 10.1093/ofid/ofab134 PMC7989186 35141343

[5] Massachusetts Department of Elementary and Secondary Education. SY22 Legacy K-12 Testing Program. https://www.doe.mass.edu/covid19/testing/legacy.html. (2022).

[6] Massachusetts Department of Elementary and Secondary Education. (Department of Public Health, 8/11/2021).

[7] Massachusetts Department of Elementary and Secondary Education. *COVID-19 Testing Program*, https://www.doe.mass.edu/covid19/testing/ (2021).

[8] Pollock, N. R. et al. Implementation of SARS-CoV2 Screening in K–12 Schools Using In-School Pooled Molecular Testing and Deconvolution by Rapid Antigen Test. *J. Clin. Microbiol.* **59**, e01123–01121 (2021). 34191585 10.1128/JCM.01123-21 PMC8373013

[9] Schechter-Perkins, E. M. et al. A test-to-stay modified quarantine program for COVID-19 in schools. *Pediatrics* **149**, e2021055727 (2022). 10.1542/peds.2021-055727 35132435

[10] Boston News. (2022).

[11] Massachusetts Department of Elementary and Secondary Education. *COVID-19 Information and Resources*, https://www.doe.mass.edu/covid19/ (2022).

[12] Massachusetts Department of Public Health. Massachusetts Department of Public Health COVID-19 Dashboard. *Dashboard Pub. Health Indicators.* 31, (2020).

[13] National Center for Education Statistics. *Data and Tools*, https://nces.ed.gov/datatools/ (2022).

[14] Massachusetts Department of Elementary and Secondary Education. Original K-12 Testing Program. https://www.doe.mass.edu/covid19/testing/original.html (2022).

[15] Massachusetts Department of Elementary and Secondary Education. 2021-22 Enrollment by Grade Report (District). (11/18/2021). https://profiles.doe.mass.edu/statereport/enrollmentbygrade.aspx.

[16] Bureau of Infectious Diseease and Laboratory Sciences. Massachusetts COVID-19 vaccination data and updates. https://www.mass.gov/info-details/massachusetts-covid-19-vaccination-data-and-updates.

[17] Bureau of Infectious Disease and Laboratory Sciences. COVID-19 Response Reporting. https://www.mass.gov/info-details/covid-19-response-reporting#covid-19-county-level-data-reporting- (2022).

[18] Tayyar, R. et al. Low infectivity among asymptomatic patients with a positive severe acute respiratory coronavirus virus 2 (SARS-CoV-2) admission test at a tertiary care center, 2020-2022. *Infect Control Hosp Epidemiol*, 1-3, 10.1017/ice.2023.210 (2023). 10.1017/ice.2023.210 PMC10910729 37746805

[19] Ferretti, L. et al. Digital measurement of SARS-CoV-2 transmission risk from 7 million contacts. *Nature* **626**, 145–150 (2024). 38122820 10.1038/s41586-023-06952-2 PMC10830410

[20] CDC Newsroom. *CDC Expands Booster Shot Eligibility and Strengthens Recommendations for 12-17 Year Olds*, https://www.cdc.gov/media/releases/2022/s0105-Booster-Shot.html (2022).

[21] Chemaitelly, H. et al. Covid-19 Vaccine Protection among Children and Adolescents in Qatar. *N. Engl. J. Med.*10.1056/NEJMoa2210058 (2022). 36322837 10.1056/NEJMoa2210058 PMC9644642

[22] Tan, S. T. et al. Infectiousness of SARS-CoV-2 breakthrough infections and reinfections during the Omicron wave. *Nat. Med.*10.1038/s41591-022-02138-x (2023). 10.1038/s41591-022-02138-x PMC9974584 36593393

[23] Ertem, Z. (Harvard Dataverse, 2024).

