# Supplementary material for: Impacts of testing and immunity acquired through vaccination and infection on covid-19 cases in Massachusetts elementary and secondary students
Source: Commun Med (Lond). 2024 Oct 16;4:202. doi: 10.1038/s43856-024-00619-3 (PMC11484689; doi:10.1038/s43856-024-00619-3)
Supplement: Supplementary file 1 — Supplementary Materials [file 43856_2024_619_MOESM1_ESM.pdf]

Supplementary Materials:

Title: Impacts of Testing and Immunity Acquired Through Vaccination and Infection on COVID-19 Cases in Massachusetts Elementary and Secondary Students

Authors: Branch-Elliman, Westyn; Ertem, Zeynep; Nelson, Richard; Danesharasteh, Anseh; Berlin, David; Fisher, Lloyd; Schechter-Perkins, Elissa.

Item	Page
**Supplementary Table 1.** Sources of Data	**2**
**Supplementary Table 2.** Biweekly Vaccination and Incidence Rates	**3**
**Supplementary Table 3.** Biweekly Testing Program Participation	**4**
**Supplementary Table 4.** Matched Retrospective Observational Cohort with a Target trial Emulation Approach, All Test Positivity Rate Modeled as the Outcome. Results analyzed using GEE models.	**5**
**Supplementary Table 5.** Random Effects Regression, Pooled Testing Positivity Rate**,** Stratified by Omicron versus Delta Periods	**6**
**Supplementary Table 6.** Random Effects Regression, All Test Positivity Rate**,** Stratified by Omicron versus Delta Periods	**7**
**Supplementary Table 7.** Random Effects Regression, Total Test Positivity, Sensitivity Analysis (First 6 weeks of the program excluded)	**8**
**Supplementary Table 8.** Impact of High versus Low Vaccination Rates in Schools during the Second Half of the Academic Year (Omicron Period), All Test Positivity	**9**
**Supplementary Figure 1.** Correlation Heatmap between covariates used to assess testing program impacts. Correlations calculated using Pearson correlation	**10**
**Supplementary Figure 2.** County-Level Incidence Rates	**11**
**Supplementary Figure 3.** In-School Testing and Positivity Rates, County-level Vaccination and Incidence Rates	**12**
**Supplementary Figure 4.** Student Consent Rates, In-School Testing and Positivity Rates	**13**
**Supplementary Figure 5.** Community and School Test Positivity Rate Trends	**14**
**Supplementary Figure 6.** Testing Positivity Rate among Elementary Schools with High versus Low Vaccination Rates	**15**
**Supplementary Figure 7.** Testing Positivity Rate among High Schools with High versus Low Vaccination Rates	**16**

Supplementary Table 1. Sources of Data

Variable	Data Source
**Testing consent rate**	CIC data file
**Test type (TTS, pooled testing, other)**	CIC data file
**Test result**	CIC data file
**Age of person receiving test**	CIC data file; estimated based on birth year
**School age of student**	Estimated based on birth year<br>*Age 5-11: Elementary school*<br>*Age 12-14: Middle school*<br>*Age 15-19: High school*<br>*Age >19: Non-student*
**School population**	DESE, NCES
**Community COVID-19 incidence rate**	Massachusetts DPH COVID-19 Dashboard
**Community COVID-19 vaccination rate**	Massachusetts DPH COVID-19 Dashboard
**COVID-19 vaccination rate (district-level)**	Estimated based on town vaccination rates from the Massachusetts DPH COVID-19 Dashboard<br>*Age 5-11: Elementary school*<br>*Age 12-14: Middle school*<br>*Age 15-19: High school*<br>*Age >19: Non-student*
**School Type (Public, private, charter, elementary, middle, high, mixed)**	DESE data file<br>NCES data file
**School Demographics (E.g., racial distribution of students, %non-Hispanic white, Title I status)**	NCES data file
**Urban-Rural Index**	DESE data file<br>NCES data file
**Pooled testing positivity rate**	Calculated variable:<br># positive pools/consented population/school/biweekly period<br><br>Note: *Due to the nature of pooled testing results, which included multiple individuals in one testing run, age of the individual was not available for these tests. For the purposes of the analysis, all positive pools were attributed to cases in students, however, some of the positive pools were likely from staff members.*
**Total testing positivity rate**	Calculated variable:<br># positive tests/consented population/school/biweekly period<br><br>Note: *Reflex testing results excluded from the analysis, as these tests were duplicates to confirm pooled testing positivity rates.*<br><br>Note: *All tests performed in individuals 19 years of age or younger were assumed to be students and all tests performed in individuals older than 19 years of age were assumed to be not students. In cases where the age variable was not available, tests were assumed to have been performed on students.*

Supplementary Table 2. Biweekly Vaccination and Incidence Rates and In-School Testing Positivity Rates in all counties

Week	County Vaccination Rate	Mean County COVID-19 Incidence Rate	Sum of County COVID-19 Incidence Rate (per 100,000 residents)	Surveillance Testing Positivity Rate* (%)	Test-to-Stay Positivity Rate* (%)	All Positive Tests	All Tests
**9/5/2021**	0.63	293.85	4114	0.79	3.75	20	1,714
**9/19/2021**	0.64	390.73	5470	0.94	0.34	129	12,473
**10/3/2021**	0.65	264.70	3706	0.80	0.33	325	35,434
**10/17/2021**	0.65	233.51	3269	0.99	0.63	565	50,398
**10/31/2021**	0.66	235.73	3300	0.85	0.61	845	86,392
**11/14/2021**	0.67	288.70	4042	1.36	1.00	1,436	96,763
**11/28/2021**	0.68	442.26	6192	2.18	0.95	1,870	109,547
**12/12/2021**	0.69	834.84	11688	3.02	1.00	4,205	192,792
**12/26/2021**	0.70	1098.95	15385	3.74	1.22	4,704	197,720
**1/9/2022**	0.71	3240.52	45367	18.33	5.47	13,065	96,261
**1/23/2022**	0.71	2725.70	38160	10.86	3.66	20,975	252,845
**2/6/2022**	0.72	1031.04	14435	4.19	1.90	7,005	162,696
**2/20/2022**	0.73	339.70	4756	1.69	1.50	2,182	97,460
**3/6/2022**	0.73	222.19	3111	0.99	0.94	536	40,743
**3/20/2022**	0.73	127.25	1781	0.81	1.08	771	70,284
**4/3/2022**	0.73	186.38	2609	1.28	1.94	1,273	71,478
**4/17/2022**	0.74	242.65	3397	2.10	1.65	2,006	74,867
**5/1/2022**	0.74	491.34	6879	2.41	1.49	1,396	45,913
**5/15/2022**	0.74	700.78	9811	4.43	2.41	5,392	97,867
**5/29/2022**	0.74	493.52	6909	4.50	2.25	5,048	92,671
**6/12/2022**	0.74	414.29	5800	2.76	2.62	2,440	67,630
**6/26/2022**	0.74	275.29	3854	2.49	3.16	825	23,799

*Calculated based on the consented population within a school, not the total school population.

Supplementary Table 3. Biweekly Population Participating in School Testing Program (Number of Consented Students, Number of Students Tested, Number of Test-to-Stay Tests Performed, Number of Pooled Testing Tests Performed, Pooled Testing Positivity Rate).

Date	School Population in the Program*	Consented Students in the Program**	Number of Students Tested	Population Consented (19 and Under) (%)	Test to Stay Tests Performed	Test to Stay Tests Positive (%)	Pooled Testing Tests Performed	Pooled Tests Positive (%)
**9/5/2021**	24,999	8,140	1,233	0.38	152	3.95	658	1.06
**9/19/2021**	164,432	49,890	10,057	0.36	3,122	0.35	6,273	0.92
**10/3/2021**	411,131	138,093	28,439	0.37	6,055	0.35	21,156	0.85
**10/17/2021**	647,539	225,993	40,006	0.39	10,430	0.61	27,611	0.98
**10/31/2021**	844,894	315,554	70,618	0.42	32,692	0.63	33,864	0.83
**11/14/2021**	861,811	341,656	80,195	0.44	40,442	0.99	34,949	1.49
**11/28/2021**	930,980	381,829	92,846	0.46	64,129	0.96	23,848	2.36
**12/12/2021**	1,002,446	446,988	165,499	0.49	112,731	1.00	42,198	3.22
**12/26/2021**	1,001,572	480,473	169,510	0.53	128,176	1.21	31,865	3.96
**1/9/2022**	972,873	513,201	76,449	0.58	32,886	5.63	28,627	19.26
**1/23/2022**	1,022,493	574,316	209,195	0.61	108,793	3.73	73,819	11.29
**2/6/2022**	977,730	578,216	129,348	0.64	46,706	1.95	67,676	4.29
**2/20/2022**	870,360	521,943	72,456	0.65	8,960	1.58	55,384	1.69
**3/6/2022**	711,810	440,458	29,446	0.67	1,178	0.68	25,234	0.91
**3/20/2022**	810,665	491,812	52,681	0.65	2,222	1.22	43,718	0.81
**4/3/2022**	799,038	492,629	53,054	0.66	2,509	1.91	42,576	1.17
**4/17/2022**	809,691	496,686	55,979	0.66	4,639	1.53	42,533	2.01
**5/1/2022**	725,924	452,623	33,283	0.67	3,141	1.43	25,068	2.39
**5/15/2022**	884,643	540,910	74,622	0.66	11,102	2.43	46,733	4.46
**5/29/2022**	858,695	529,924	70,700	0.67	9,160	2.33	45,673	4.51
**6/12/2022**	747,025	467,942	51,198	0.68	3,508	2.65	39,961	2.83
**6/26/2022**	467,116	305,970	17,980	0.69	1,021	3.23	14,418	2.47

*Total population from all schools participating in in-school testing programs on the corresponding week.

** Population of students consented to the program on the corresponding week.

Supplementary Table 4. Matched Retrospective Observational Cohort with a Target trial Emulation Approach, All Test Positivity Rate Modeled as the Outcome. Results analyzed using GEE models.

	Elementary	Middle	High	Combined Program
**Surveillance testing program (yes/no)**	-0.01* [-0.02, -0.002]	-0.01 [-0.03, 0.012]	-0.02 [-0.05, 0.005]	-0.02* [-0.04, -0.0001]
**Cases per 100,000**	0.000001 [-0.0000001, 0.000002]	0.000004** [0.000001, 0.00001]	0.000002 [-0.000000001, 0.000004]	0.000003** [0.000001, 0.000004]
**Vaccination (School-level)**	-0.01 [-0.04, 0.027]	-0.04* [-0.08, -0.011]	-0.01 [-0.03, 0.015]	-0.005 [-0.04, 0.029]

** p<0.01, * p<0.05. Week 0 is 9/19/2024. Variables used for propensity matching (average vaccination, consent rate, race, urban/rural, title1 status, charter, and public/private status) not included in the regression models. For the propensity adjustment, average vaccination rate for the whole population was used. Models are adjusted for age-stratified vaccination rates and program week. Wald test was used to evaluate statistical significance. Variables in bold are statistically significant to p <0.05.

Supplementary Table 5. Random Effects Regression Stratified by School Type and Circulating variant, Pooled Testing Positivity Rate Modeled as the Outcome

		**Elementary Schools (N=706)**		**Middle Schools (N=222)**		**High Schools (N=253)**		**Mixed School Age (N=392)**	
		β-coefficient	p-value	β-coefficient	p-value	β-coefficient	p-value	β-coefficient	p-value
Delta (Until Dec 15)	County Cumulative Cases per 100,000 residents	**0.467**	**0.000**	**0.696**	**0.000**	**0.400**	**0.000**	**0.451**	**0.000**
	Proportion of school-aged children fully vaccinated in the district*	-	-	**0.038**	**0.000**	**0.008**	**0.003**	**-0.014**	**0.000**
	Consent rate	**0.021**	**0.000**	**0.017**	**0.000**	**0.020**	**0.000**	**0.014**	**0.000**
	Student Demographics								
	African American (%)	-0.0001	0.083	-0.0001	0.414	-0.00001	0.795	**-0.0001**	**0.001**
	Hispanic (%)	**-0.0002**	**0.000**	-0.0001	0.165	-0.0001	0.177	**-0.0001**	**0.000**
	White (%)	-0.00001	0.810	0.0001	0.335	0.00004	0.167	**0.0001**	**0.002**
	Other	**0.0001**	**0.001**	**0.0004**	**0.000**	0.0001	0.231	0.00002	0.492
	Rural Status	**0.005**	**0.000**	0.005	0.065	**0.006**	**0.000**	**0.011**	**0.000**
	School Demographics								
	Title1 Status	**-0.002**	**0.001**	-0.001	0.360	-0.002	0.094	-0.002	0.086
	Charter	**0.010**	**0.009**	**0.018**	**0.000**	**-0.007**	**0.005**	**0.003**	**0.017**
	Public	**0.002**	**0.002**	**0.004**	**0.022**	**0.006**	**0.000**	**0.004**	**0.000**
Omicron (Dec 16 to June)	County Cumulative Cases per 100,000 residents	**-0.402**	**0.000**	**-0.521**	**0.000**	**-0.466**	**0.000**	**-0.434**	**0.000**
	Proportion of school-aged children fully vaccinated in the district*	**-0.069**	**0.000**	**-0.062**	**0.000**	**-0.021**	**0.001**	**-0.088**	**0.000**
	Consent rate	**-0.014**	**0.000**	**-0.015**	**0.001**	**-0.031**	**0.000**	**-0.007**	**0.031**
	Student Demographics								
	African American (%)	-0.00004	0.493	-0.0001	0.557	**0.0003**	**0.008**	0.0001	0.131
	Hispanic (%)	0.0001	0.290	0.0002	0.125	**0.0002**	**0.023**	-0.00001	0.809
	White (%)	0.0001	0.165	-0.0001	0.620	0.00005	0.558	**-0.0001**	**0.004**
	Other	**0.0002**	**0.000**	-0.0002	0.228	-0.00004	0.701	-0.00005	0.439
	School Demographics								
	Rural Status	**-0.011**	**0.000**	-0.005	0.200	**-0.009**	**0.013**	-0.001	0.711
	Title1 Status	-0.002	0.210	0.002	0.474	0.001	0.785	-0.0003	0.915
	Charter Status	0.006	0.431	**-0.023**	**0.013**	**0.011**	**0.048**	-0.002	0.627
	Public	**-0.004**	**0.011**	-0.006	0.074	**-0.015**	**0.000**	**-0.008**	**0.000**

Supplementary Table 6. Random Effects Regression, Stratified by School Type and Circulating Variant, All Test Positivity Rate Modeled as the Outcome

		Elementary Schools		Middle Schools		High Schools		Multiple Programs	
Delta (Until Dec 15)	Cumulative cases per 100,000 county residents	**0.116**	**0.000**	**0.295**	**0.000**	**0.434**	**0.000**	**0.052**	**0.000**
	Fully vaccinated	-	-	**0.017**	**0.000**	**0.015**	**0.000**	**-0.009**	**0.000**
	Consent rate	**0.007**	**0.000**	**0.012**	**0.000**	**0.021**	**0.000**	**0.004**	**0.000**
	Demographics of Students								
	African American	-0.000005	0.463	**-0.00003**	**0.059**	-0.00004	0.142	-0.00001	0.462
	Hispanic	**-0.00004**	**0.000**	**-0.0001**	**0.000**	**-0.0001**	**0.000**	**-0.00003**	**0.000**
	White	-0.00001	0.239	**-0.00005**	**0.000**	**-0.0001**	**0.002**	0.000004	0.584
	Other	**0.00002**	**0.001**	**0.0001**	**0.000**	**0.0001**	**0.000**	0.000004	0.754
	Demographics of Schools								
	Rural Status	**0.001**	**0.000**	**0.002**	**0.000**	**0.006**	**0.000**	0.001	0.085
	Title 1 Status	**-0.0004**	**0.001**	-0.0001	0.905	**-0.002**	**0.025**	-0.001	0.329
	Charter School	0.001	0.289	**0.006**	**0.000**	**-0.006**	**0.001**	-0.0001	0.929
	Public School	**0.001**	**0.000**	**0.002**	**0.002**	**0.006**	**0.000**	0.0002	0.659
Omicron (Dec 16 to June)	Cumulative cases per 100,000 county residents	**-0.093**	**0.000**	**-0.023**	**0.000**	**-0.146**	**0.000**	**-0.235**	**0.000**
	Fully vaccinated	**-0.023**	**0.000**	**-0.007**	**0.000**	**-0.010**	**0.000**	**-0.046**	**0.000**
	Consent rate	**-0.006**	**0.000**	-0.00003	0.534	**-0.014**	**0.000**	**-0.003**	**0.028**
	Demographics of Students								
	African American	**-0.00005**	**0.021**	0.00005	0.142	0.0001	0.141	0.00004	0.110
	Hispanic	-0.00001	0.468	0.00003	0.417	0.00002	0.510	-0.00003	0.159
	White	0.00003	0.123	0.0001	0.200	0.00004	0.211	**-0.0001**	**0.001**
	Other	**0.0001**	**0.000**	0.002	0.295	-0.0001	0.221	**-0.0001**	**0.055**
	Demographics of Schools								
	Rural Status	**-0.004**	**0.000**	-0.0004	0.690	-0.001	0.401	**-0.004**	**0.014**
	Title 1 Status	-0.001	0.065	-0.002	0.649	0.001	0.358	**-0.003**	**0.040**
	Charter School	**0.007**	**0.026**	-0.0004	0.740	0.005	0.082	**0.004**	**0.023**
	Public School	**-0.001**	**0.050**	**-0.023**	**0.000**	**-0.005**	**0.000**	0.0001	0.956

Variables in bold are significant to p<0.05.

Supplementary Table 7. Sensitivity Analysis: First 6 Weeks of the Program Excluded (Pooled Testing Positivity Rate Modeled Outcome)

		Elementary Schools		Middle Schools		High Schools		Mixed School Age	
	Positivity Rate	β-coefficient	p-value	β-coefficient	p-value	β-coefficient	p-value	β-coefficient	p-value
Delta (Until Dec 15)	County Cumulative Cases per 100,000 residents	**0.508**	**0.000**	**0.678**	**0.000**	**0.447**	**0.000**	**0.466**	**0.000**
	Proportion of school-aged children fully vaccinated in the district	-	-	**0.037**	**0.000**	**0.012**	**0.001**	**-0.009**	**0.000**
	Consent rate	0.025	0.000	**0.019**	**0.000**	**0.021**	**0.000**	**0.016**	**0.000**
	Demographics of Students								
	African American	-0.0001	0.060	-0.00004	0.554	0.00001	0.924	**-0.0001**	**0.045**
	Hispanic	**-0.0002**	**0.000**	-0.0001	0.168	-0.00005	0.278	**-0.0001**	**0.000**
	White	-0.00003	0.505	0.00003	0.654	0.0001	0.191	**0.0001**	**0.002**
	Other	**0.0001**	**0.002**	**0.0004**	**0.000**	0.0001	0.106	0.00003	0.280
	Demographics of Schools								
	Rural status	**0.006**	**0.000**	0.005	0.126	**0.006**	**0.001**	**0.010**	**0.000**
	Title1 status	**-0.003**	**0.004**	-0.001	0.616	-0.003	0.152	-0.001	0.305
	Charter school	**0.013**	**0.011**	**0.017**	**0.001**	-0.007	0.029	**0.003**	**0.052**
	Public school	**0.002**	**0.014**	**0.004**	**0.035**	**0.007**	**0.000**	**0.005**	**0.000**
Omicron (Dec 16 to June)	County Cumulative Cases per 100,000 residents	**-0.402**	**0.000**	**-0.521**	**0.000**	**-0.466**	**0.000**	**-0.434**	**0.000**
	Proportion of school-aged children fully vaccinated in the district	**-0.069**	**0.000**	**-0.062**	**0.000**	**-0.021**	**0.001**	**-0.088**	**0.000**
	Consent rate	**-0.014**	**0.000**	**-0.015**	**0.001**	**-0.031**	**0.000**	**-0.007**	**0.031**
	Demographics of students								
	African American	-0.00004	0.493	-0.0001	0.557	**0.0003**	**0.008**	0.0001	0.131
	Hispanic	0.0001	0.290	0.0002	0.125	**0.0002**	**0.023**	-0.00001	0.809
	White	0.0001	0.165	-0.0001	0.620	0.00005	0.558	**-0.0001**	**0.004**
	Other	**0.0002**	**0.000**	-0.0002	0.228	-0.00004	0.701	-0.00005	0.439
	Demographics of School								
	Rural status	**-0.011**	**0.000**	-0.005	0.200	**-0.009**	**0.013**	-0.001	0.711
	Title 1 status	-0.002	0.210	0.002	0.474	0.001	0.785	0.000	0.915
	Charter school	0.006	0.431	**-0.023**	**0.013**	**0.011**	**0.048**	-0.002	0.627
	Public school	**-0.004**	**0.011**	-0.006	0.074	**-0.015**	**0.000**	**-0.008**	**0.000**

Variables in bold are significant to $p<0.05$

Supplementary Table 8. Impact of high versus low vaccination rates in schools during the second half of the academic year (Omicron period), All Test Results Modeled Outcome

		**Elementary School**				**High School**			
	Positivity Rate	β-coefficient	p-value	Confidence Interval		β-coefficient	p-value	Confidence Interval	
Jan 1st – Feb 28th	County Cumulative Cases per 100,000 residents	**-1.205**	**0.000**	**-1.225**	**-1.186**	**-1.463**	**0.000**	**-1.510**	**-1.416**
	Consent rate	**-0.055**	**0.000**	**-0.060**	**-0.051**	**-0.056**	**0.000**	**-0.064**	**-0.047**
	Demographics of students								
	African American	**0.0001**	**0.053**	**0.000**	**0.000**	**0.0004**	**0.000**	**0.000**	**0.001**
	Hispanic	**0.001**	**0.000**	**0.001**	**0.001**	**0.001**	**0.000**	**0.001**	**0.001**
	White	-0.00002	0.732	0.000	0.000	**0.0004**	**0.000**	**0.000**	**0.001**
	Other	**-0.0004**	**0.000**	**0.000**	0.000	**-0.0003**	**0.009**	**-0.001**	**0.000**
	Demographics of school								
	Rural status	**-0.011**	**0.000**	**-0.015**	-0.008	**-0.018**	**0.000**	**-0.027**	**-0.010**
	Title 1 status	**0.003**	**0.028**	**0.000**	0.005	**0.017**	**0.000**	**0.010**	**0.024**
	Charter school	**0.015**	**0.045**	**0.000**	0.029	0.001	0.840	-0.011	0.014
	Public school	**-0.005**	**0.001**	**-0.007**	-0.002	**-0.029**	**0.000**	**-0.036**	**-0.023**
	Low vaccination uptake* (yes/no)	**0.016**	**0.000**	**0.013**	0.019	**0.031**	**0.000**	**0.024**	**0.037**
Mar 1st – End	County Cumulative Cases per 100,000 residents	**0.209**	**0.000**	**0.199**	**0.219**	**0.437**	**0.000**	**0.406**	**0.468**
	Consent rate	**0.005**	**0.000**	**0.003**	**0.007**	**0.011**	**0.000**	**0.006**	**0.016**
	Demographics of students								
	African American	-0.00001	0.951	0.000	0.000	-0.0001	0.228	0.000	0.000
	Hispanic	**-0.0001**	**0.000**	**0.000**	**0.000**	**-0.0002**	**0.000**	**0.000**	**0.000**
	White	0.00002	0.294	0.000	0.000	0.00001	0.909	0.000	0.000
	Other	-0.00002	0.527	0.000	0.000	**0.0001**	**0.026**	**0.000**	**0.000**
	Demographics of school								
	Rural status	**0.002**	**0.005**	**0.001**	**0.004**	**0.007**	**0.001**	**0.003**	**0.012**
	Title1 status	**-0.002**	**0.001**	**-0.003**	**-0.001**	**-0.005**	**0.007**	**-0.009**	**-0.001**
	Charter school	0.0002	0.927	-0.005	0.006	-0.0002	0.947	-0.007	0.006
	Public school	**0.002**	**0.006**	**0.000**	**0.003**	**0.010**	**0.000**	**0.006**	**0.014**
	Low vaccination uptake* (yes/no)	**-0.003**	**0.000**	**-0.004**	**-0.002**	**-0.011**	**0.000**	**-0.014**	**-0.007**

*Defined as <50% of students fully vaccinated against COVID-19 at a town level.

Variables in bold are significant to the p<0.05 level.

Supplementary Figure 1. Correlation Heatmap between covariates used to assess testing program impacts. Correlations calculated using Pearson correlation.

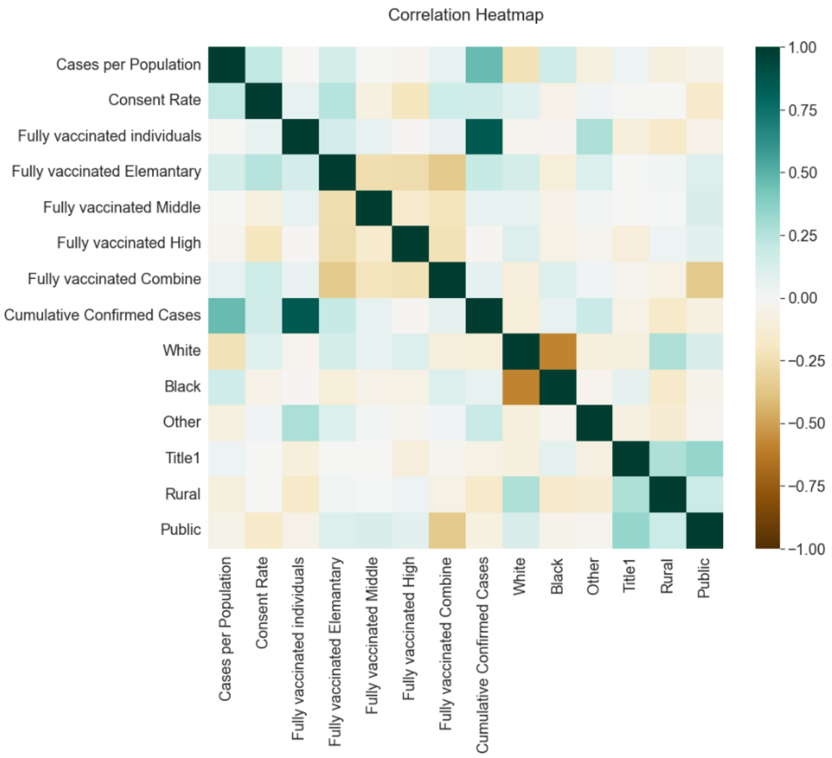

Supplementary Figure 2. County-Level Incidence Rates

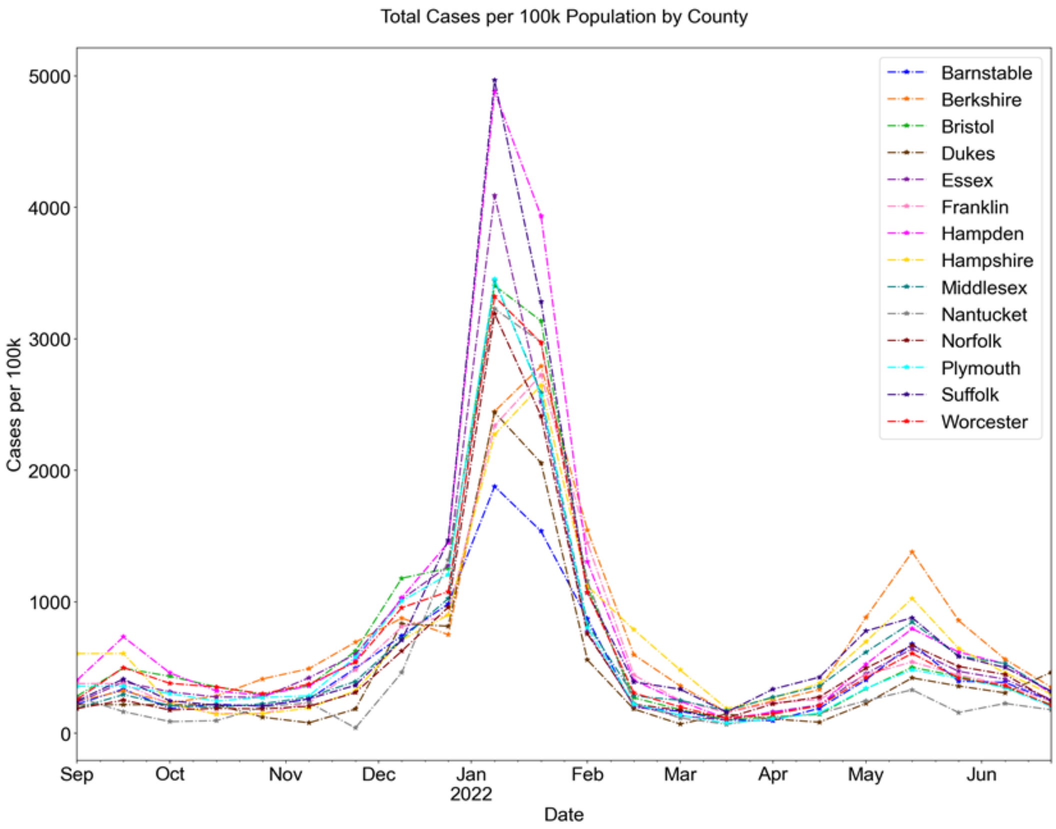

Supplementary Figure 3. In-School Testing and Positivity Rates, County-level Vaccination and Incidence Rates

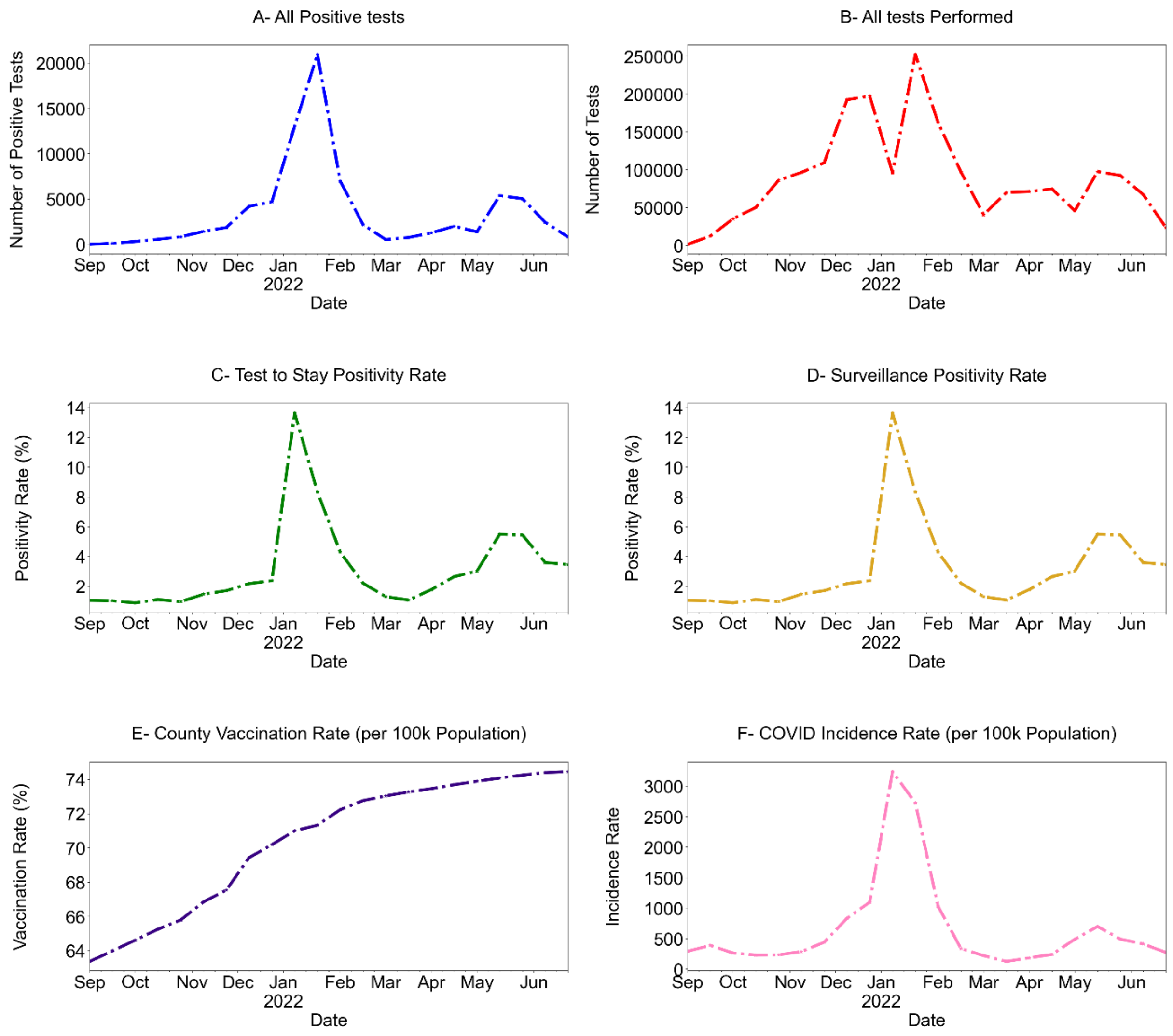

A- Sum of all positive tests by all tests; B- Number of all tests that performed; C- Sum of all positive test to stay tests by all test to stay test results; D- Sum of all positive surveillance tests by all surveillance test results; E- County-level vaccination rate per 100k residents; F- COVID-19 Incidence rate per 100k county residents

Supplementary Figure 4. Student Consent Rates, In-School Testing and Positivity Rates

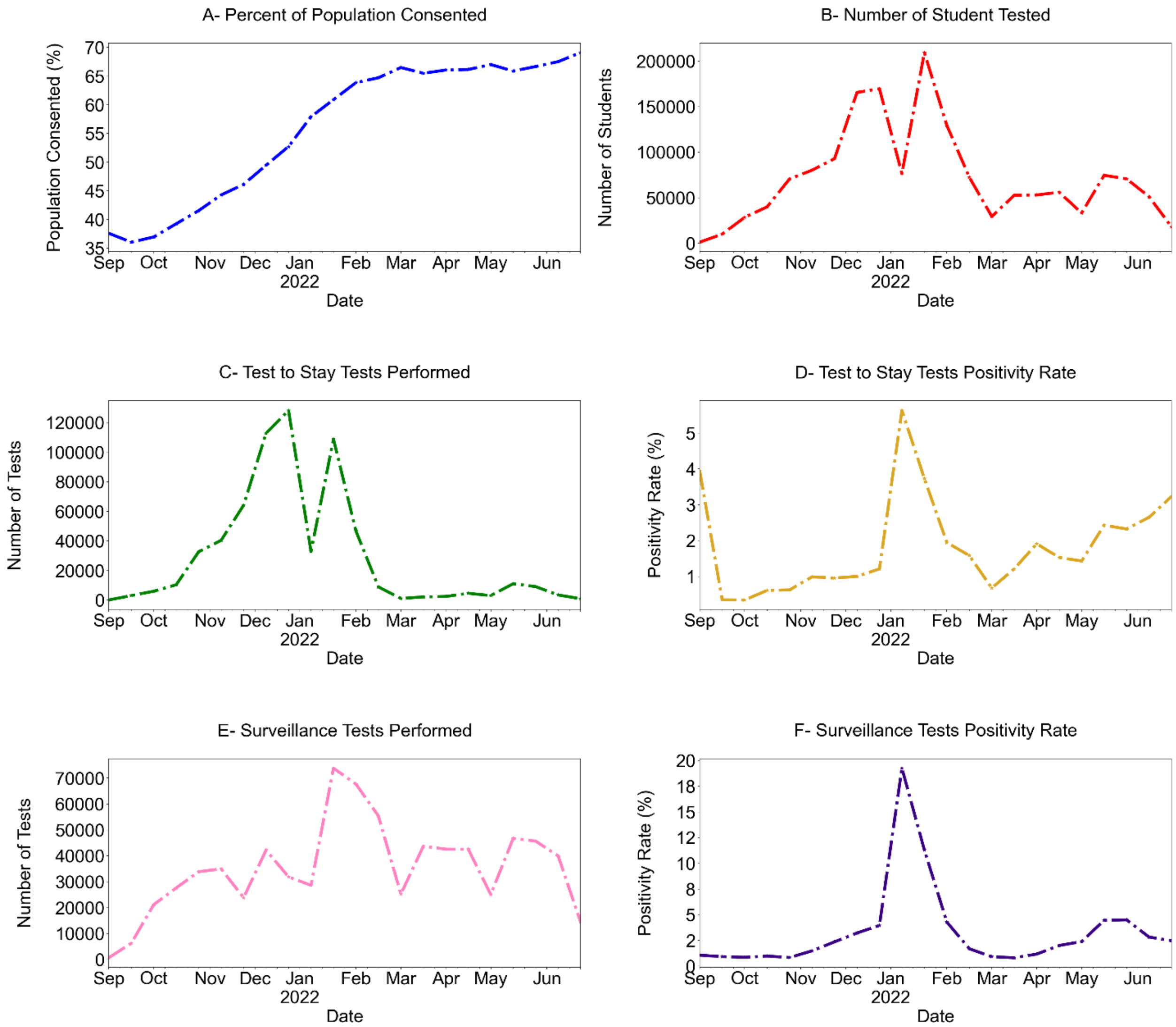

A- Percentage of students consented to the program; B- Total nimber od students tested; C- Total number of test to stay tests performed; D- Test to stay positivity rate is calculated as sum of all positive test to stay tests by sum of all test o stay test results; E- All surveillance tests performed; F- Surveillance tests positivity rate is calculated as sum of all positive surveillance tests by sum of all surveillance test results

Supplementary Figure 5. Community and school test positivity rate trends

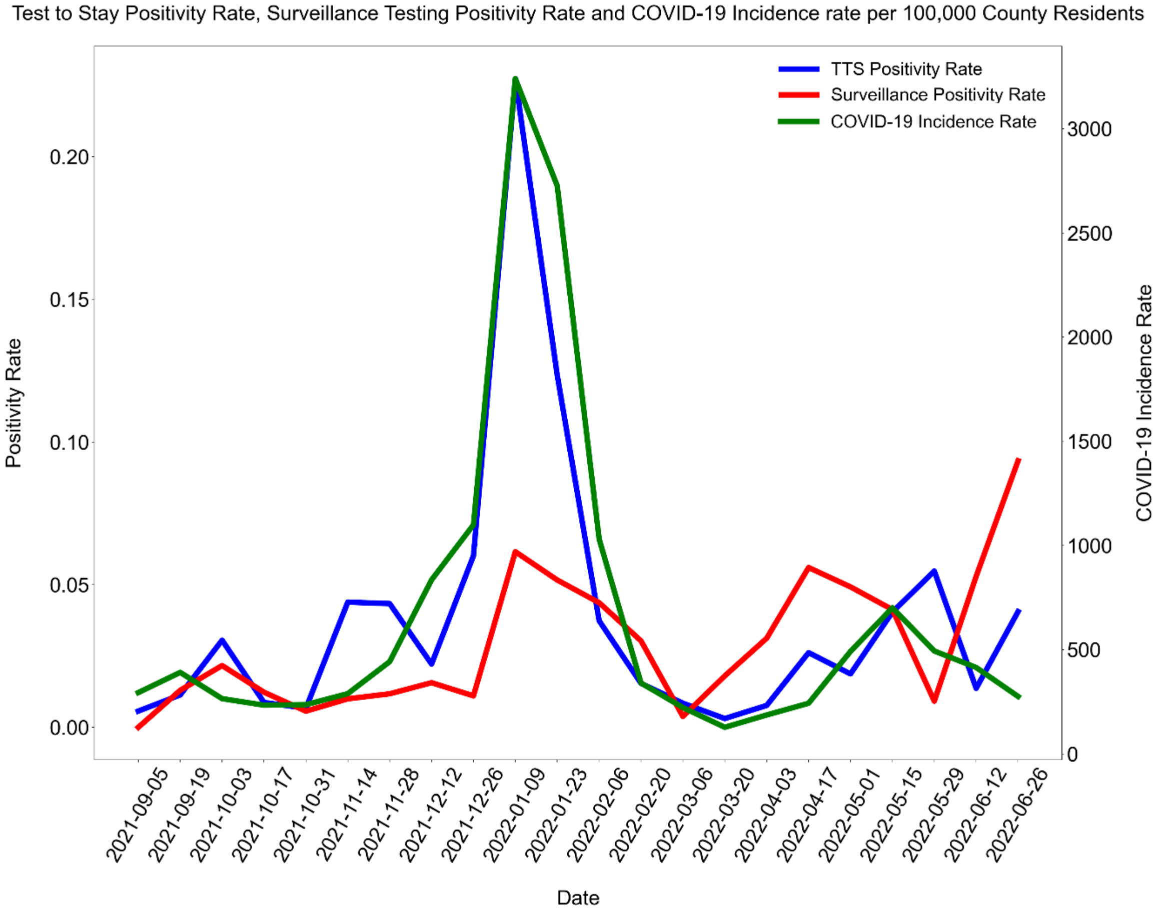

Supplementary Figure 6. Testing Positivity Rate among Elementary Schools with High versus Low Vaccination Rates. *Panel A*: All Test Results. *Panel B*: Pooled Testing Positivity.

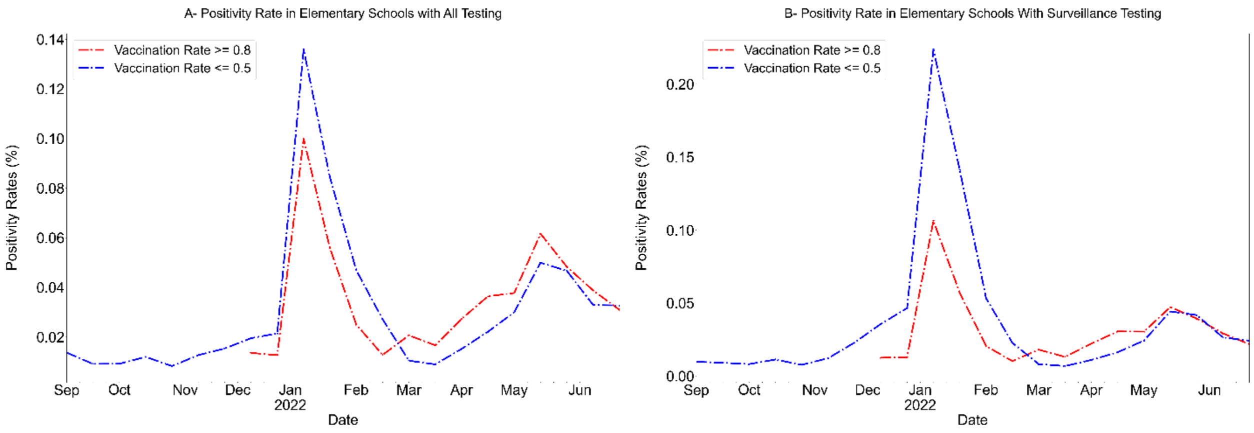

A and B- Blue line indicates positivity rates for elementary school students with more than 80% vaccination rate and red line indicates positivity rates for elementary school students with less than 50% vaccination rate

Supplementary Figure 6 Legend. (A) Unadjusted means of all tests positivity rate in elementary schools with low vaccination rate (less than 50%) and high vaccination rate (higher than 80%). (B) Unadjusted means of surveillance test positivity rate in elementary schools with low vaccination rate (less than 50%) and high vaccination rate (higher than 80%). Blue lines represent schools with <50% vaccination rates and red lines represent schools with >80% vaccination rates.

Supplementary Figure 7. Testing Positivity Rate among High Schools with High versus Low Vaccination Rates. *Panel A*: All Test Results. *Panel B*: Pooled Testing Positivity.

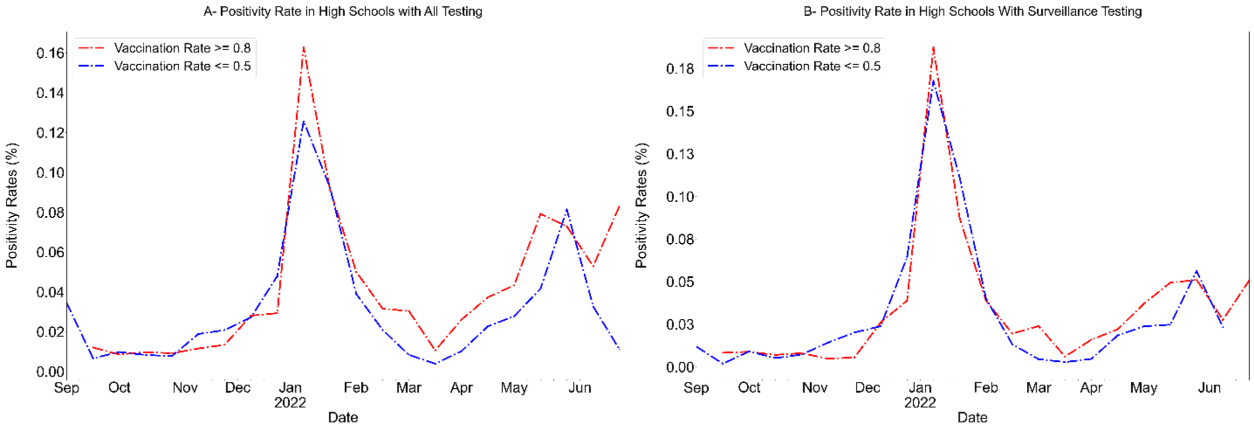

A and B- Blue line indicates positivity rates for high school students with more than 80% vaccination rate and red line indicates positivity rates for high school students with less than 50% vaccination rate

Supplementary Figure 7 Legend. A) Unadjusted means of all tests positivity rate in high schools with low vaccination rate (less than 50%) and high vaccination rate (higher than 80%). (B) Unadjusted means of pooled testing positivity rate in High Schools with low vaccination rate (less than 50%) and high vaccination rate (higher than 80%). The red line represents schools with a ≥ 80% vaccination rate and the blue line represents schools with ≥ 50% vaccination rate.
